# On Spasm or Cramp of the Stomach

**Published:** 1829-10-01

**Authors:** 


### XXVII.

### ON SPASM OR CRAMP OF THE STOMACH. By Dr. MACFARLANE.

We are fully convinced that some of the sudden and unexplained deaths which we so often hear of, are occasioned by the complaint at the head of this paper, though overlooked by the great majority of systematic writers. If we can judge by the painful evidence of our own senses or the descriptions which patients sometimes draw of their own feelings, this cramp of the stomach is one of the most dreadful species of suffering, while it lasts, to which humanity is exposed. Fortunately it is of short duration, when violent in degree. Life indeed is incompatible with any long duration of the spasm. But in these cases, the complaint is by no means confined to the stomach. We are convinced that the principal seat of the spasm is the *diaphragm*, and that this location, or, at all events, participation in the complaint, occasions the dreadful difficulty, or rather the dread of breathing, which characterises the higher grades of gastric cramp. The complaint is quite a different thing from gastrodynia, or dyspepsia—though dyspeptic symptoms are often the forerunners or accompaniments of the spasms. Dr. Macfarlane has published an interesting paper on this subject in our Glasgow contemporary, for May, 1829, of which we shall take some notice here. The following is the Doctor's concise description of the attack.

"When spasm affects the stomach, the morbid excitement is communicated through the medium of the nerves to the muscular tissue, inducing the most acute pain, with a feeling of rigid contraction, violent twisting or tearing in the epigastrium, soon followed by painful and interrupted breathing, difficult articulation, pallid countenance, small, hurried, and contracted pulse, and occasionally with coldness of the extremities, and rigid contraction of the recti abdominis and gastrocnemii muscles. Such symptoms will, in general, present themselves during a well-marked paroxysm of this disease."

The complaint, in dyspeptic subjects, is generally called forth by eating cold or indigestible substances, and often goes off without attaining much violence. But gastro-diaphragmatic spasm not seldom occurs where there has been no previous stomach-complaint; and where the general health has been merely deranged by confinement, sedentary employment, or the depressing passions, or great fatigue. We shall glance at some of the cases which Dr. M. has adduced as examples of this painful malady.

*Case* 1. This was a man, about 40 years of age, who had suffered from long imprisonment in Glasgow Jail, and was suddenly seized with spasm in the stomach. He complained of pain in the epigastrium darting to the left false ribs and umbilicus. The abdominal muscles in these parts felt rigid, and strong pressure afforded relief. He tossed about in great agony, pressing the epigastrium with his clenched fist—his face was pale, anxious, and covered with perspiration—pulse quick and small—pain so acute as to impede his utterance. A drachm of sulphuric ether, with 50 drops of laudanum, removed the spasm in ten minutes. Dr. M. has often prescribed this formula in similar cases with manifest advantage, but sometimes a repetition of the dose is necessary.

"In severe forms of the disease, the patient usually complains of a sensation of rigid contraction or drawing together in the epigastric region, occasioned by the inordinate contraction of the muscular coat of the stomach, and occasionally producing a hard circumscribed tumour perceptible to touch. When, however, the abdominal muscles participate in the spasm, the tension and inequality of surface produced by the morbid contraction of the recti abdominis, effectually prevent the discovery of this tumour. The diaphragm, it is presumed, very soon sympathizes with this state of the stomach, and becomes also spasmodically affected, as the short, interrupted, and highly distressed respiration, and the difficult articulation evidently show. Indeed, every person who has seen a violent attack of this complaint, must have observed the change in the respiration which takes place at the height of the paroxysm; the difficulty, and often the impossibility of performing inspiration and expiration, in an unobstructed manner, and the half suppressed cries or moans which the patient utters, apparently occasioned by the rigidly contracted diaphragm, remaining as an almost immoveable partition between the thorax and abdomen. If the hand is applied either to the thorax or epigastrium, we can seldom discover the alternate elevations and depressions of these parts, indicative of a natural state of breathing."

*Case* 2. Our author was called late at night to a gentleman who had been taken suddenly ill. He complained of excruciating pain in the centre of the epigastrium—his face was pallid and contracted—his expression wild and remarkably altered in physiognomy—extremities cold—pulse 130 and feeble—utterance very difficult on account of the pain. A full dose of ether and opium was given, and warm fomentations were applied to the epigastrium. He experienced relief for ten minutes; but the pain and spasms returned with increased intensity. His cries for relief were incessant—the epigastrium became tense and prominent—and pressure rather aggravated his sufferings. Another dose of opium was given with ammonia; but no good effects followed. Dr. Brown was added in consultation. The breathing had become laborious—he vomited some of the medicine, which appeared as if tinged with blood, and the same coloured fluid was squirted from the mouth and nostrils. He still pointed to the stomach as the seat of pain. The body was cold, the pulse fluttering. He expired before midnight, that is, in less than an hour from the commencement of medical assistance, and in about eight hours from the beginning of the pain. Unfortunately the body was not permitted to be opened, and therefore the cause of death is problematical. The Doctor imagines that the stomach was lacerated during the violence of the spasm. We are inclined to think that either an ulcer had existed previously in the stomach, which gave way at this time, or that an aneurism had burst, and produced the fatal event. We once witnessed nearly a similar train of symptoms produced by the bursting of an aneurism within the chest, and destroying life in twelve hours.

*Case* 3. A poor weaver, aged 22 years, was suddenly seized, at 8 o'clock in the evening, (February 26th,) with excruciating pain at the pit of the stomach. He could scarcely articulate, and kept beating the epigastrium with his fist. A glass of whiskey was immediately given him, and this produced momentary relief; but in a few minutes the pain was as bad as ever. By 2 o'clock in the morning his agony was insupportable. He fainted; and soon afterwards threw up a dark brown fluid, like moss water. He was now visited by a surgeon, who considered the disease to be spasm, and bled him, exhibiting afterwards a draught of valerian and assafœtida, followed by a cathartic. At 3 o'clock, p.m. of the 27th February, Dr. Macfarlane saw him. He was lying on his back, with his legs drawn up, face pale, breathing quick, pulse feeble and 140, skin cold and clammy. He still complained of constant and severe pain in the epigastric region, and now diffused over the abdomen, rendering pressure intolerable. On the right side of the epigastrium there was a small tumour obscurely defined, and presenting indistinct fluctuation. He continued to vomit a dark coloured fluid, which was thrown from his mouth in a stream about the thickness of a quill, like water from a small syringe. He expired at 8 o'clock that evening, 24 hours from the commencement of his illness.

" *Dissection.* The cavity of the abdomen contained several pints of a dark-coloured fluid, which had a strong smell of assafœtida. The stomach was sunk and almost empty, being nearly covered by a distended colon. On grasping it, fluid was seen to issue through a longitudinal opening in its anterior surface, between the greater and lesser curvatures, about three inches from the pylorus. Ligatures were passed around the termination of the œsophagus and commencement of the duodenum, and the stomach was carefully removed for more minute inspection. It contained an ounce of fluid and a little undigested food. Its coats were throughout of a natural thickness and appearance, except the peritoneal covering, which had several large patches of a bright red colour on its surface. The mucous tissue was neither inflamed, ulcerated, nor pulpy. The perforation readily admitted the finger, and extended from above downwards; its edges were carefully examined with a glass, and found slightly ragged, but without thickening, ulceration, or any other morbid appearance in the neighbouring textures. There was obviously no loss of substance where the opening existed, for its edges could be brought together without producing a folding, or puckering of the adjoining parts."

Dr. M. is inclined to think that, in this case, the stomach was rent or torn by the violence of the spasm, and that the peritoneal inflammation was a sequence. This conjecture is very probable.

*Case.* 4. This case proves that the disease in question may prove fatal without leaving any cognizable trace in the dead body.

A lady, 28 years of age, was affected about twelve hours after her first confinement, with well-marked symptoms of spasm in the stomach, and that without any apparent cause.

" She had taken a little arrow-root prepared with water, and had slept for several hours, when she was suddenly awakened by excruciating pain in the region of the stomach. She struck this part with her hand, pressed upon it with all her force, and complained of great difficulty of breathing. The pulse was small and rapid, the skin cold and moist, and the countenance of a deadly paleness. Two draughts containing full doses of laudanum, æther, and volatile tincture of valerian, were exhibited, without effect; sinapisms, prepared with turpentine, were applied to the stomach and inferior extremities; and about half an hour before death, when the powers of life were rapidly sinking, brandy was freely given. On dissection, the only morbid appearance that could be discovered by the most accurate investigation, was general softening of the cerebellum, with vascular turgescence in the base of the brain."

We return our thanks to Dr. M. for the perusal of this interesting paper; and have only to add that, when the existence of spasm is once ascertained, the stimulants should not be sparingly administered. It is astonishing with what facility a spasmed stomach will bear diffusible stimuli and strong narcotics. The quantities then taken, with impunity, would, in health, set up inflammation in that organ, and stupify the brain and nervous system.

